# Supplementary material for: R-Spondin2, a Positive Canonical WNT Signaling Regulator, Controls the Expansion and Differentiation of Distal Lung Epithelial Stem/Progenitor Cells in Mice
Source: Int J Mol Sci. 2022 Mar 13;23(6):3089. doi: 10.3390/ijms23063089 (PMC8954098; doi:10.3390/ijms23063089)
Supplement: Supplementary file 1 [file ijms-23-03089-s001.zip › SuppFigures 1-5.pdf]

Supplement Figures

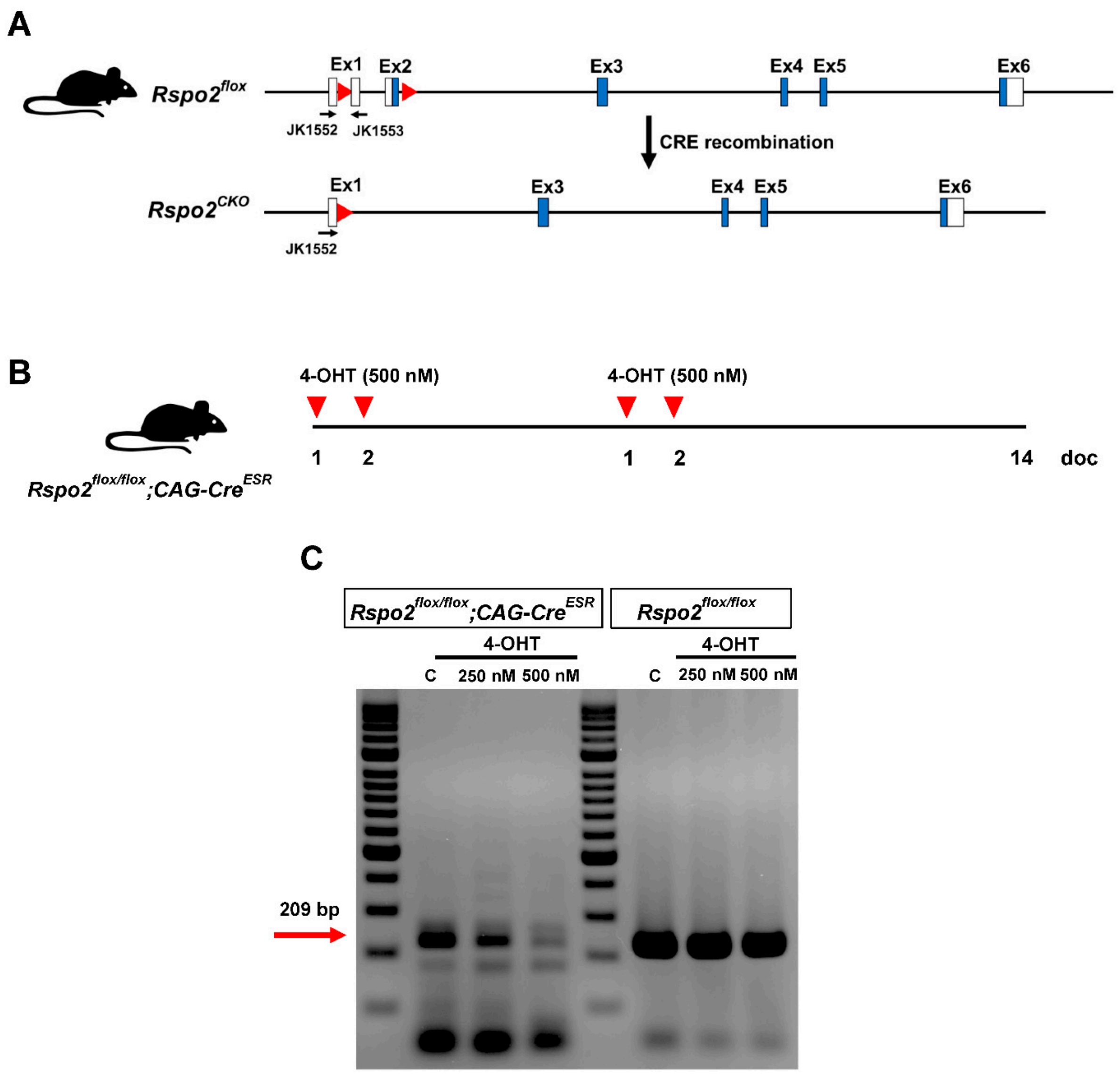

Figure S1. (A) Schematic structure of conditional *Rspo2* knockout allele before (*Rspo2*^{flox}) and after (*Rspo2*^{CKO}) 4-OHT-induced CRE recombination. (B) Schematic experimental schedule for conditional *Rspo2* knockout. (C) PCR using genomic DNA to confirm CRE recombination efficacy on the Rspo2^{flox} allele after 4-OHT treatment.

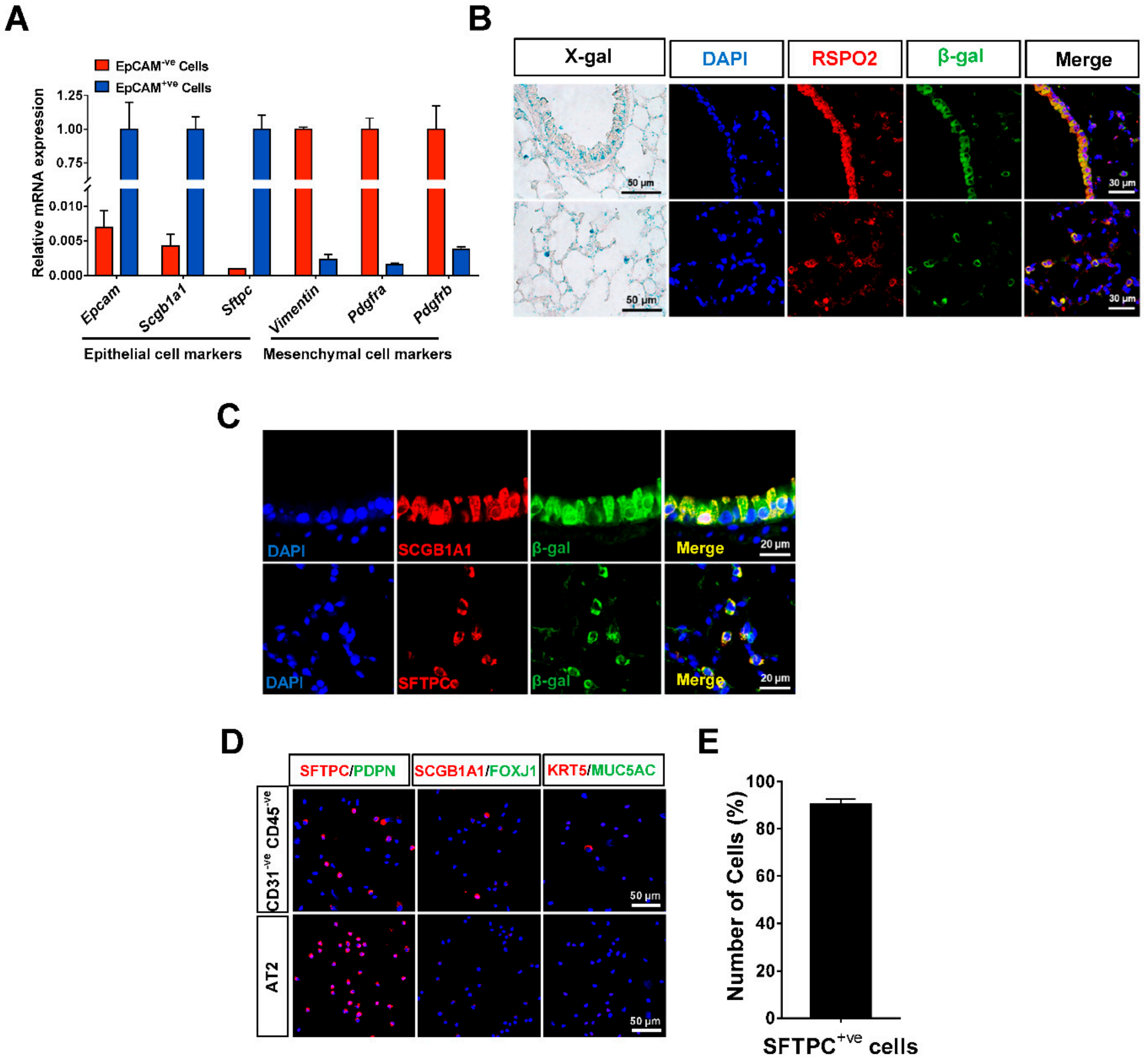

Figure S2. (A) qRT-PCR analysis of the expression of epithelial and mesenchymal cell marker genes in isolated DLESPs. (B) X-gal histochemical staining and immunofluorescence staining of RSPO2 and β-galactosidase (β-Gal) in the lungs of *Rspo2*$^{LacZ/+}$ mice. (C) Immunofluorescence staining of β-Gal and club cell marker, SCGB1A1, or AT2 cell marker, SFTPC, in *Rspo2*$^{LacZ/+}$ mouse lung tissues. (D) Immunofluorescence staining of different epithelial cell markers in isolated CD31^{-ve} CD45^{-ve} whole lung cells and AT2 cells. (E) Quantification of SFTPC^{+ve} cells among the isolated AT2 cells. Data are presented as mean ± SEM.

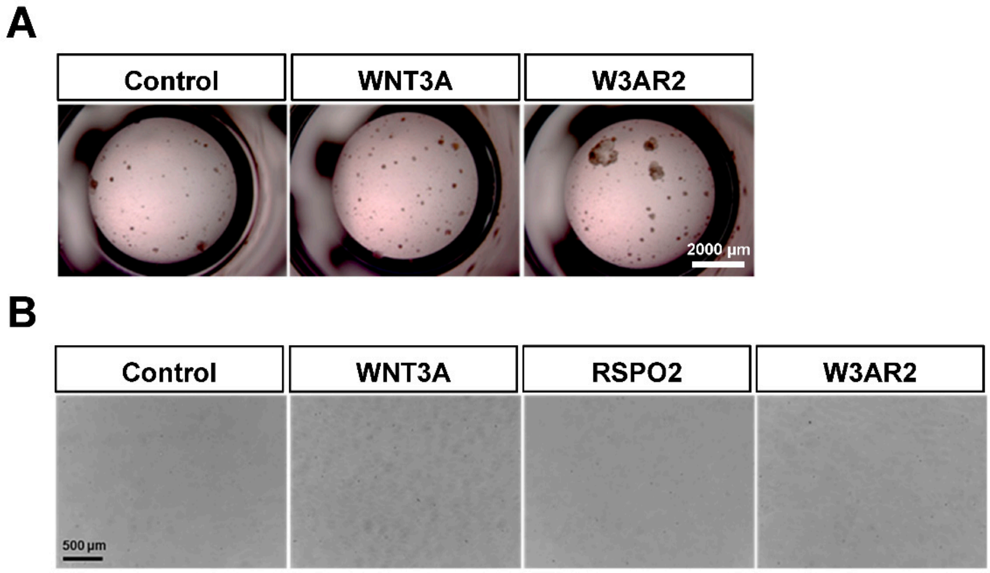

Figure S3. (A) Bright-field images of DLESP-derived organoid cultures treated with WNT3A (20 ng/ml) or W3AR2 (WNT3A, 20 ng/ml + RSPO2, 200 ng/ml). (B) Bright-field images of DLESP-derived organoids treated with WNT3A (20 ng/ml), RSPO2 (200 ng/ml), and both (W3AR2) in fibroblast-free organoid cultures.

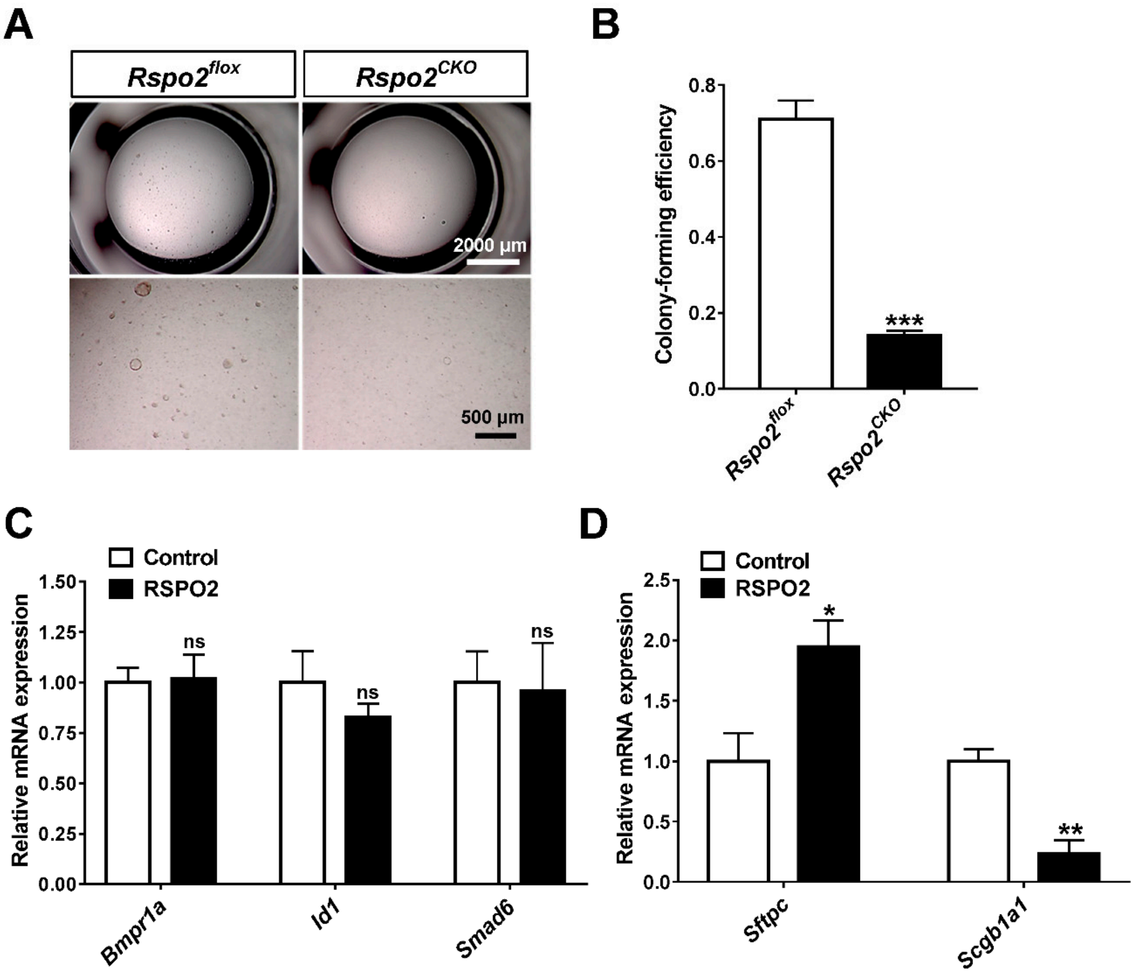

Figure S4. (A and B) Colony formation of *Rspo2*^{CKO} and control *Rspo2*^{flox} club cells in fibroblast-free, FGF10/HGF supplemented 3D organoid cultures. (C) qRT-PCR analysis of *Sftpc* and *Scgb*1a1 in DLESP organoids treated with RSPO2 (200 ng/ml). (D) qRT-PCR analysis of BMP downstream target genes, Bmpr1a, *Id1*, and *Smad6*, in club cell-derived organoids. All organoids were harvested at day14 of culture for further analysis. Data are presented as mean ± SEM. ns: no statistical significance, *$p < 0.05$, **$p < 0.01$, ***$p < 0.001$.

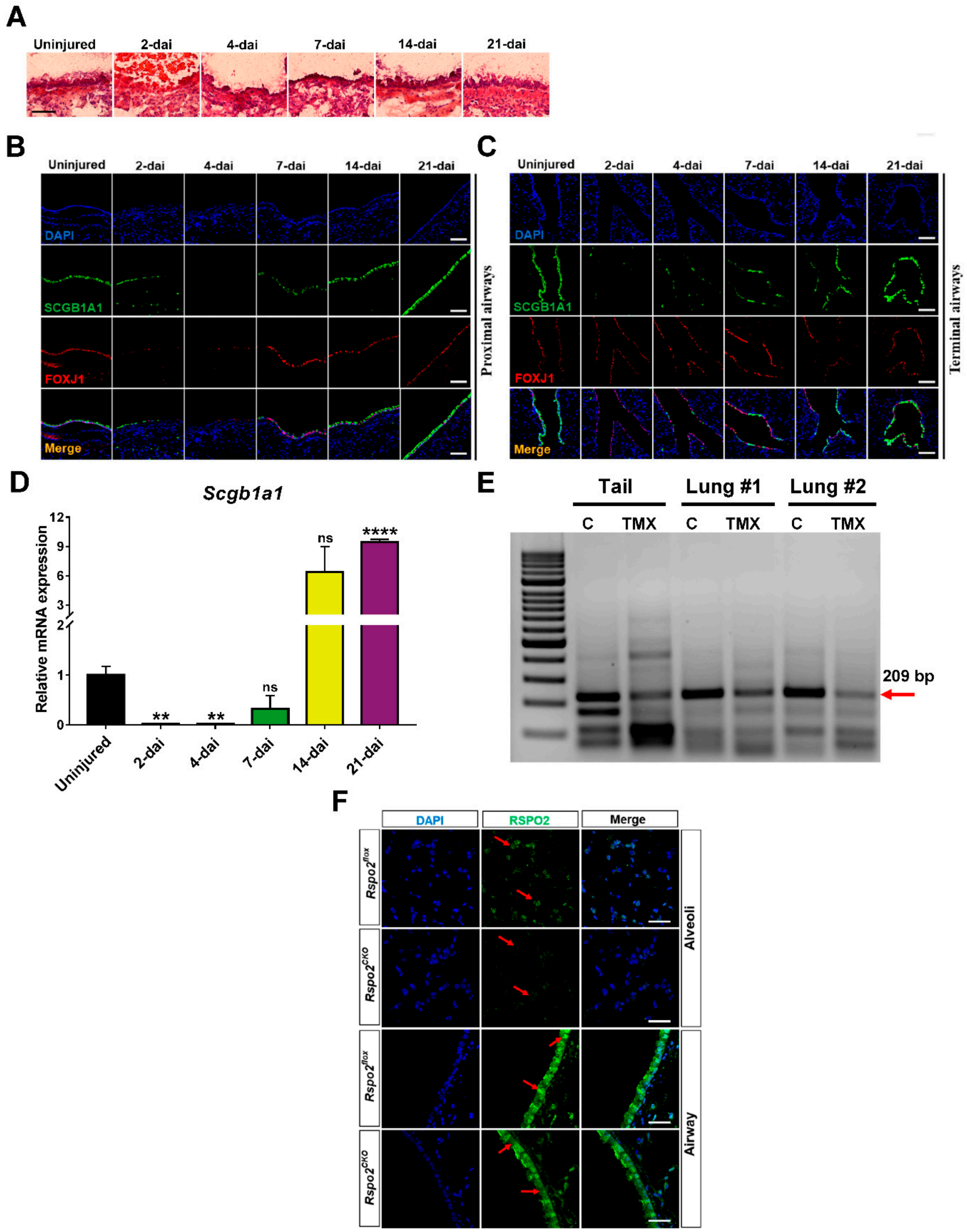

Figure S5. (A) Hematoxylin and eosin staining of regenerating distal airway tissues after naphthalene-induced acute lung injury. Dai, days after injury; scale bar, 100 µm. (B and C) Immunofluorescence staining of SCGB1A1 and FOXJ1 in club and ciliated cells during airway regeneration. Scale bar, 100 µm. (D) qRT-PCR analysis

of *Scgb1a1* expression during regeneration. (E) PCR using genomic DNA to confirm the efficiency of conditional *Rspo2* knockout after tamoxifen (TMX) or corn oil (C) injection in mice. (F) Immunofluorescence staining of RSPO2 in *Rspo2*^{CKO} and control *Rspo2*^{flox} lung tissues. Scale bar, 30 μm. Data are presented as mean ± SEM. ns: no statistical significance, **p < 0.01, ****p < 0.0001.
