# Supplementary material for: R-Spondin2, a Positive Canonical WNT Signaling Regulator, Controls the Expansion and Differentiation of Distal Lung Epithelial Stem/Progenitor Cells in Mice
Source: Int J Mol Sci. 2022 Mar 13;23(6):3089. doi: 10.3390/ijms23063089 (PMC8954098; doi:10.3390/ijms23063089)
Supplement: Supplementary file 1 [file ijms-23-03089-s001.zip › SuppTable S1.pdf]

Supplementary Table S1. List of antibodies used for immunofluorescence staining.

Primary antibodies	Types	Dilution	Supplier	Catalog #
Mouse anti-Ac-α-tubulin	Monoclonal, IgG2b	1:500	Sigma-Aldrich	T6793
Mouse anti-FOXJ1	Monoclonal, IgG1	1:250	Thermo Fisher Scientific	14-9965-82
Mouse anti-HOPX	Monoclonal, IgG1	1:200	Santa Cruz	SC-398703
Rabbit anti-Ki-67	Rabbit, IgG	1:200	Cell signaling technology	9129
Rabbit anti-KRT5	Polyclonal, IgG	1:500	BioLegend	905504
Rabbit anti-MUC5AC	Monoclonal, IgG1	1:200	Thermo Fisher Scientific	MA5-12178
Syrian hamster anti-PDPN	Monoclonal, IgG	1:250	Thermo Fisher Scientific	14-5381-82
Mouse anti-RSPO2	Monoclonal, IgG1	1:1500	Yoon Lab	
Rabbit anti-non-phosphorylated-β-catenin (Active-β-catenin)	Rabbit, IgG	1:1000	Cell signaling technology	8814
Rabbit anti-RSPO2	Rabbit, IgG	1:500	Proteintech	17781-1-AP
Rabbit anti-SCGB1A1	Rabbit, IgG	1:500	Abcam	ab40873
Rabbit anti-SFTPC	Rabbit, IgG	1:500	EMD Millipore	ABC99
Mouse anti-β-galactosidase	Monoclonal, IgG1	1:500	Sigma-Aldrich	G8021
Mouse anti-β-galactosidase	Monoclonal, IgM	1:500	Sigma-Aldrich	G6282
**Secondary antibodies**		**Dilution**	**Supplier**	**Catalog #**
Alexa Fluor 488 F(ab')$_2$ fragment donkey anti-mouse IgG (H+L)		1:750	Jackson ImmunoResearch	715-546-150
Alexa Fluor 488 goat anti-syrian hamster IgG (H+L)		1:750	Jackson ImmunoResearch	107-545-142
Cy3 donkey anti-mouse IgG (H+L)		1:750	Jackson ImmunoResearch	715-165-150
Cy3 F(ab')$_2$ fragment donkey anti-rabbit IgG (H+L)		1:750	Jackson ImmunoResearch	711-166-152
Cy3 goat anti-rabbit IgG (H+L)		1:750	Jackson ImmunoResearch	111-165-003
FITC donkey F(ab')$_2$ fragment anti-mouse IgM		1:750	Jackson ImmunoResearch	715-096-020
FITC goat anti-mouse IgG (H+L)		1:750	Jackson ImmunoResearch	115-095-166
