# Supplementary material for: R-Spondin2, a Positive Canonical WNT Signaling Regulator, Controls the Expansion and Differentiation of Distal Lung Epithelial Stem/Progenitor Cells in Mice
Source: Int J Mol Sci. 2022 Mar 13;23(6):3089. doi: 10.3390/ijms23063089 (PMC8954098; doi:10.3390/ijms23063089)
Supplement: Supplementary file 1 [file ijms-23-03089-s001.zip › SuppTable S2.pdf]

Supplementary Table S2. DNA sequences of oligonucleotide primers

Gene	Direction	Sequence (5'-3')	Application
Abca3	Forward	AGC AGG ACC GGG AGT CTA AG	qRT-PCR
	Reverse	ACA GTG GAG GGA TAG TGA GCA	
Actb	Forward	ATG GAG GGG AAT ACA GCC C	qRT-PCR
	Reverse	TTC TTT GCA GCT CCT TCG TT	
Aqp5	Forward	ATT GGC TTG TCG GTC ACA CT	qRT-PCR
	Reverse	ACG ATC GGT CCT ACC CAG AA	
Axin2	Forward	TGA CTC TCC TTC CAG ATC CCA	qRT-PCR
	Reverse	TGC CCA CAC TAG GCT GAC A	
Bmpr1a	Forward	AAC AGC GAT GAA TGT CTT CGA G	qRT-PCR
	Reverse	GTC TGG AGG CTG GAT TAT GGG	
Bpifa1	Forward	TGC CTT TGG CTG TAA GCC C	qRT-PCR
	Reverse	AGA ATT GCC TCC TCC AGA CTT TA	
Cyp2f2	Forward	GGA CCC AAA CCT CTC CCA ATC	qRT-PCR
	Reverse	CCG TGA ACA CCG ACC CAT AC	
Epcam	Forward	GCG GCT CAG AGA GAC TGT G	qRT-PCR
	Reverse	CCA AGC ATT TAG ACG CCA GTT T	
Foxj1	Forward	ACC AAG ATC ACT CTG TCG GC	qRT-PCR
	Reverse	TCT CCC GAG GCA CTT TGA TG	
Gapdh	Forward	AGA AGA CTG TGG ATG GCC CCT C	qRT-PCR
	Reverse	GAT GAC CTT GCC CAC AGC CTT	
Id1	Forward	CCT AGC TGT TCG CTG AAG GC	qRT-PCR
	Reverse	CTC CGA CAG ACC AAG TAC CAC	
Krt5	Forward	TCT GCC ATC ACC CCA TCT GT	qRT-PCR
	Reverse	CCT CCG CCA GAA CTG TAG GA	
Lgr5	Forward	AGC CTT CAA TCC CTG CGC CTA G	qRT-PCR
	Reverse	GAC AGG GAC GTC TGT GAG AGC ATT G	
Muc5ac	Forward	GTG GTT TGA CAC TGA CTT CCC	qRT-PCR
	Reverse	CTC CTC TCG GTG ACA GAG TCT	
Pdgfra	Forward	TCC ATG CTA GAC TCA GAA GTC A	qRT-PCR
	Reverse	TCC CGG TGG ACA CAA TTT TTC	

pdgfrb	Forward	TTC CAG GAG TGA TAC CAG CTT	qRT-PCR
	Reverse	AGG GGG CGT GAT GAC TAG G	
Pdpn	Forward	GGG ATG AAA CGC AGA CAA CAG	qRT-PCR
	Reverse	TTA GGG CGA GAA CCT TCC AG	
Rspo2	Forward	ATG TGC ACG ATG CAG AAT AGA	qRT-PCR
	Reverse	ACC AAC TTC ACA ACC TTC TAC	
Rtkn2	Forward	GTC TGT TCA CGG AGA TGA GG	qRT-PCR
	Reverse	AGC CAA CCA GAC CTT TTC C	
Scgb1a1	Forward	ATG AAG ATC GCC ATC ACA ATC AC	qRT-PCR
	Reverse	GGA TGC CAC ATA ACC AGA CTC T	
Sftpa	Forward	GAG GAG CTT CAG ACT GCA CTC	qRT-PCR
	Reverse	AGA CTT TAT CCC CCA CTG ACA G	
Sftpc	Forward	GAG GTC CTG ATG GAG AGT CC	qRT-PCR
	Reverse	CCA CGA TGA GAA GGC GTT TG	
Smad6	Forward	GCA ACC CCT ACC ACT TCA GC	qRT-PCR
	Reverse	GTG GCT TGT ACT GGT CAG GAG	
Tubb4a	Forward	CAC CTG CAA GCC GGT CAA T	qRT-PCR
	Reverse	TCC CCA TGA TAG GTC CCA GTG	
Vimentin	Forward	CGG CTG CGA GAG AAA TTG C	qRT-PCR
	Reverse	CCA CTT TCC GTT CAA GGT CAA G	
**JK1552**	Forward	GTA GGC GAT CTA CAC CTG GTG CTG	Genomic DNA PCR
**JK1553**	Reverse	CAA CCT CTG CGG AGC ACT GCC TTC	
